# The Role of Osteocytes in Inflammatory Bone Loss

**DOI:** 10.3389/fendo.2019.00285

**Published:** 2019-05-14

**Authors:** Corinne E. Metzger, S. Anand Narayanan

**Affiliations:** ^1^Department of Health and Kinesiology, Texas A&M University, College Station, TX, United States; ^2^Department of Medical Physiology, Texas A&M Health Science Center, Bryan, TX, United States

**Keywords:** osteocyte, cytokines, inflammation, tumor necorosis factor (TNF), sclerostin

## Abstract

Osteoimmunology investigations to-date have demonstrated the significant interactions between bone surface cells, osteoclasts and osteoblasts, and immune cells. However, there is a paucity of knowledge on osteocytes, cells embedded in the bone matrix, and their role in inflammation and inflammatory bone loss. Osteocytes communicate through various mechanisms; directly via dendritic processes and through secretion of proteins that can influence the formation and activity of osteoblasts and osteoclasts. Some osteocyte proteins (e.g., interleukin-6 and RANKL) also have roles within the immune system. In the context of mechanical loading/unloading, the regulatory role of osteocytes is well understood. More recent data on osteocytes in various inflammatory models suggest they may also aid in orchestrating inflammation-induced changes in bone turnover. In inflammatory conditions, osteocytes express multiple pro-inflammatory cytokines which are associated with increases in bone resorption and declines in bone formation. Cytokines are known to also influence cell population growth, maturation, and responsiveness via various signaling modalities, but how they influence osteocytes has not been greatly explored. Furthermore, osteocytes may play regulatory roles in orchestrating bone's response to immunological changes in inflammatory conditions. This review will address what is known about osteocyte biology in physiological conditions and in response to varying immunological conditions, as well as highlight key areas of interest for future investigations.

Bone continually adapts to its internal and external environment, undergoing formation and resorption to maintain bone mass. Bone loss is an imbalance of bone formation and resorption which occurs in many conditions including disuse, aging, and chronic inflammatory conditions such as inflammatory bowel disease (1), rheumatoid arthritis (2), psoriasis (3), and systemic lupus erythematosus (4). While comparatively less studied to disuse or age-related bone loss, inflammatory conditions are also associated with increased fracture incidence (4–8). Multiple other conditions with known inflammatory components are also associated with bone loss and increased bone fragility including type 2 diabetes (9), chronic kidney disease (10), spinal cord injury (11), and aging-related osteoporosis (12). While the role of immune factors and inflammation on various bone cells (osteoblasts, osteoclasts, stromal cells, marrow immune cells, bone precursor cells) have been extensively researched (13), the osteocyte response to inflammatory stimuli has been comparably less investigated. However, osteocytes have increasingly become appreciated as key regulators of both osteoclasts and osteoblasts, orchestrating changes in bone turnover.

## Interactions of the Immune System and Bone

The immune system interacting with bone physiology is being elucidated in the burgeoning field of osteoimmunology. For example, cytokines are signaling proteins released by various cell-types that modulate the direction of an immune response but can also influence the cellular phenotype of immune and parenchymal cells. In general, pro-inflammatory cytokines are grouped as Th_1_, while anti-inflammatory cytokines as Th_2_ (14). Notably, cytokines can interact with bone cells leading to increased bone resorption and decreased bone formation that, over time, leads to inflammatory bone loss (15). Key Th_1_ cytokines include TNF-α and IL-1β, both stimulators of osteoclastogenesis (16–19). Additionally, these cytokines increase the production of RANKL, a key osteoclastogenesis regulator (20–22). TNF-α can also increase production of OPG, the decoy receptor for RANKL (23). IL-6 has equivocal roles in bone physiology, but, with TNF-α, IL-6 synergizes to stimulate osteoclasts and increase production of RANKL and OPG (21). IL-17 is also a potent stimulus for osteoclastogenesis (24) and sensitizes osteoclast pre-cursors to RANKL (25).

In addition to stimulating an increase in osteoclasts and bone resorption, Th_1_ cytokines interact with osteoblast development and function. For example, TNF-α inhibits osteoblast genes and differentiation factors (e.g., *RUNX2*), and reduces bone collagen synthesis (20, 26–28). TNF-α also inhibits the anabolic effects of IGF-I on osteoblasts (29) and induces osteoblast apoptosis (20, 30). Interleukin-1β (IL-1β) has been shown to also suppress bone formation (31). In addition to osteoblasts and osteoclasts, other cell types residing in the bone environment such as bone marrow stromal cells and osteoblast/osteoclast-precursor cells can be influenced in their function by cytokines (32, 33). This illustrates the critical role of immunological factors, such as Th_1_ cytokines, in bone biology.

Th_2_ cytokines, such as IL-4 and IL-10, are less well understood in the context of bone physiology. In cell culture models, both IL-4 and IL-10 have been shown to inhibit osteoclasts and reduce RANKL production (34–37). Another cell culture study found both IL-4 and IL-13 increased OPG mRNA expression; thus, decreasing RANKL mediated osteoclastogenesis *in vitro* (38). Furthermore, IL-10 transgenic knockout mice have low bone mass and increased fragility which alludes to an influential role of IL-10 in regulating bone turnover (39). There exist many other cytokines within the Th_1_ and Th_2_ classes and other subsets (Th_9_, Th_17_, Th_22_, T_fh_) that have roles not yet delineated in bone physiology, highlighting areas of future research. Finally, the interaction of these cytokines with osteocytes has been minimally investigated.

## Osteocyte Biology

Osteocytes are the longest living bone cell, making up 90–95% of cells in bone tissue in contrast to osteoclasts and osteoblasts making up ~5% (40). Osteocytes form when osteoblasts become buried in the mineral matrix of bone and develop distinct features. Residing within the lacuna of the mineralized bone matrix, osteocytes form dendritic processes that extend out from their cell bodies into spaces known as canaliculi. Through these dendritic processes, osteocytes form networks interfacing with other osteocytes, cells on bone surfaces, and the marrow (40). Through these communication networks, osteocytes sense the local and systemic environment within the bone.

Osteocytes also coordinate the actions of osteoblasts and osteoclasts via several mechanisms. First, osteocytes express and release proteins that signal to osteoblasts, osteoclasts, and other bone-residing cells to respond to environmental changes. Osteocytes express important factors for the maintenance of mineral homeostasis including SOST, Phex, DMP1, and FGF23 (41). Sclerostin, the protein encoded by the SOST gene, is an antagonist of the Wnt/β-catenin system, with increased sclerostin expression leading to a suppression of bone formation (42–44). Osteocytes also produce RANKL and OPG, critical regulators of osteoclastogenesis. While osteoblasts and other bone-residing immune cells also produce RANKL, it is now appreciated that RANKL synthesized by osteocytes is a significant source of RANKL driving osteoclast formation for bone remodeling (45–47). Additionally, osteocyte apoptosis signals to increase osteoclast activity driving targeted bone resorption (41, 48, 49). Elucidating osteocyte function in the context of osteoimmunology may provide further insight to the imbalance of resorption vs. formation seen in inflammation-induced bone loss.

## The Role of osteocytes in Adaptations to Mechanical Strains

In the past few decades, the central role of osteocytes in response to mechanical strains has been explored and identified. Osteocytes sense mechanical strains through fluid flow shear stress through the lacuna-canalicular network and changes in interstitial hydrostatic pressure (50–52). Decreased mechanical strains also induce osteocyte apoptosis leading to decreased bone mass and strength (53, 54). Some preliminary evidence suggests that high mobility group box 1 (HMGB1), an alarmin (55), may be released during osteocyte apoptosis thereby triggering RANKL and other immune factors (56). It is unknown what other immune-related factors may be released during apoptosis and the signaling cascades that follow.

Mechanosensory signals also trigger osteocytes to release various proteins that impact bone turnover. RANKL and OPG are also known to be mechanosensitive (57) and mice lacking osteocyte RANKL are protected from disuse-induced bone loss (46). Furthermore, unloading-induced osteocyte apoptosis initiates an increase in osteocyte RANKL (54). Prevention of osteocyte apoptosis in animal models of unloading mitigates increases in osteocyte RANKL (54, 58). Disuse is also characterized by elevated osteocyte sclerostin in conjunction with decreased bone formation rate (59, 60). Other mechanosensitive osteocyte proteins include insulin-like growth factor-I (IGF-I) and IL-6 which both are upregulated with loading (60–63). The role of osteocytes in the mechanosensory capabilities of bone highlight the important role these cells play in bone adaptations to the environment. Some osteocyte proteins known to have mechanosensory roles such as RANKL and IL-6 are also signaling molecules in the immune system and play key roles in inflammatory processes. This suggests that other cytokines may perhaps have similar dual roles responding under conditions of loading/unloading and under conditions of inflammation.

## Osteocytes and Cytokines

Burgeoning research has shown cytokines directly impact osteocyte apoptosis and cause the release of cytokines that influence bone turnover. In cell culture models, osteocyte apoptosis can be induced by both TNF-α and IL-1β (64–66). A mouse infectious osteomyelitis model lead to increases in osteocyte apoptosis, as well as elevations in gene expression of TNF-α, IL-1β, IL-6, and IL-17 in the femur; the same treatment in TNF-α deficient mice resulted in fewer apoptotic osteocytes (67). We previously demonstrated in rats with inflammatory bowel disease decreased osteocyte density and increased apoptosis concurrent with elevated osteocyte TNF-α (68, 69). Therefore, one mechanism of increased bone resorption in inflammatory conditions is through the direct effect of pro-inflammatory cytokines on osteocyte apoptosis which, in turn, increases osteoclastic driven resorption.

Osteocytes also express pro-inflammatory cytokines. TNF-α is expressed in the MLO-Y4 osteocyte-like cell culture line (70, 71), as is IL-6 (61). Cultured human trabecular bone chips expressing osteocyte-specific genes also express TNF-α, IL-6, IL-1β, and IL-8 (72). Other cell culture osteocyte lines have increased expression of pro-inflammatory cytokines with exposure to monosodium urate crystals (73), Brucella abortus infection (74) and orthopedic implant materials (75). Immunohistochemical analysis of rat bones demonstrate elevated osteocyte TNF-α, IL-6, and IL-17 in various inflammatory conditions (68, 69, 76). Therefore, osteocytes express cytokines that can increase osteoclastogenesis and inhibit osteoblast formation or activity.

Osteocytes themselves respond to circulating pro-inflammatory cytokines influencing their cytokine expression. For example, exposing MLO-Y4 osteocytes in culture to IL-17 increases expression of TNF-α (71). In cultured human bone chips with osteocyte-enriched cells, gene expression of TNF-α, IL-1β, and IL-6 is elevated upon treatment combinations of TNF-α, IL-1β, and IL-6 (72). Based on the supporting data from these *in vitro* studies, cytokines influence osteocytes in a positive-feedback mechanism leading to even greater cytokine expression. This would suggest osteocytes may amplify an inflammatory bone state resulting in increased production of factors altering bone turnover and increasing bone loss.

Many cytokines also alter osteocyte signaling proteins. Osteocyte-to-osteoclast signaling is enhanced by multiple pro-inflammatory cytokines largely through RANKL signaling. Culture media from IL-1β-treated MLO-Y4 cells increased osteoclastogenesis *in vitro* (77). Furthermore, blocking IL-17A prevented the increase in osteocyte RANKL due to continuous parathyroid hormone exposure (78).

MLO-Y4 cells treated with IL-6/IL-6R and co-cultured with osteoclast precursors also results in increased osteoclastogenesis due to elevated RANKL (79). Furthermore, RANKL-positive osteocytes are elevated in animal models of inflammatory conditions including periodontitis (80–82), spinal cord injury (76), and inflammatory bowel disease (68, 69). Furthermore, in rat models of inflammatory bowel disease and spinal cord injury, RANKL-positive osteocytes were associated with increases in osteoclast surfaces (68, 76). In contrast to RANKL, OPG is less well understood in conditions of inflammation. Treatment of cultured human osteocytes- with a combination of IL-1β, TNF-α, and IL-6 upregulates OPG (72). In rodent inflammatory bowel disease and spinal cord injury models, OPG-positive osteocytes were elevated (68, 69, 76). Therefore, while the exact role of OPG is not known, it is known that inflammatory cytokines regulate osteoclastogenesis in part through osteocyte-mediated RANKL/OPG signaling.

Inflammatory signals also influence osteocyte proteins controlling bone formation. Wnt proteins are key mediators of osteoblastogenesis and govern the formation of the skeletal development. Both sclerostin and Dickkopf-related-1 (Dkk-1) inhibit the Wnt signaling pathway in bone. Dkk-1 is upregulated by TNF-α and blockade against Dkk-1 in transgenic mice with inflammatory arthritis prevents bone loss (83); however, osteocyte-specific deletion of Dkk1 did not protect against inflammatory arthritis-induced bone loss (84). Interestingly, Dkk1 expression was inhibited in osteoblast cell culture treated with IL-17A; whether this is also true in osteocytes is unknown (85). Sclerostin has also been shown to bind to LRP5/6 and inhibit Wnt signaling *in vitro* and *in vivo* (42, 86), and be transcriptionally activated by TNF-α (87). In human osteocyte-enriched cell cultures, serum from rheumatoid arthritis patients, IL-1β alone, and a combination of IL-1β, TNF-α, and IL-6 all increased SOST expression (72). Furthermore, osteocyte sclerostin is elevated in animal models of high fat diets with elevated serum and osteocyte TNF-α (87), inflammatory arthritis (83), periodontitis alveolar bone (81, 82), spinal cord injury (76), and inflammatory bowel disease (68, 69). Beyond direct effects of pro-inflammatory cytokines on osteoblasts and bone formation, the inflammation-induced elevation of inhibitors of bone formation contributes to a state of low bone formation.

While outside the scope of this review, additional osteocyte proteins involved in mineral homeostasis and metabolism are influenced by inflammatory signals. Fibroblast growth factor 23 (FGF23), a phosphate regulator synthesized by osteocytes, has increased expression in inflammatory conditions (72, 88, 89). IL-17A has been shown to decrease various genes of osteocyte proteins involved in mineral metabolism including Dmp1 and Phex (90). Therefore, it is clear that osteocytes respond to inflammatory signals through various mechanisms including increased expression of cytokines and altered expression of regulatory proteins.

## Mechanosensing and Inflammatory Signals

Crucial to osteocyte function is sensing and responding to bone interstitial fluid shear stress and mechanical strains. Furthermore, there is some overlap in signaling proteins between inflammation and mechanosensing (sclerostin, RANKL, OPG, etc.). What is not fully understood is if mechanosensing is tied with osteocyte inflammatory responses and vice versa. Utilizing pulsatile fluid flow in MLO-Y4 cells, TNF-α, and IL-1β treatment inhibits fluid flow-induced increases in calcium uptake and nitric oxide release indicating a potential blunting of the osteocyte response to mechanical strains (65). Another investigation found media from MLO-Y4 cells cultured with IL-1β induced osteoclastogenesis, while IL-1β-cultured cells that also underwent pulsatile fluid flow prevented osteoclastogenesis (77). Pulsatile fluid flow reduced MLO-Y4 expression of TNF-α- and IL-17A-induced increases in TNF-α and RANKL (71), inhibited TNF-α-induced osteocyte apoptosis (66), and increased IL-6 production in osteocyte cultured cells (61).

With aging, osteocytes develop morphological adaptations and changes in the lacunocanalicular system that may impair their mechanosensory function and ability to communicate (91, 92). In addition, osteocytes of aged mice also express a senescence-associated secretory-phenotype, expressing multiple pro-inflammatory cytokines including IL-17A, IL-1A, and IL-6, likely contributing to age-related bone loss (93). Prevention of the pro-inflammatory secretome of senescent cells in aged mice with a JAK inhibitor improved bone mass and strength (94). It has been hypothesized that exercise to increase mechanical strain on bone could improve the senescent phenotype in aging (95). It remains to been seen whether a lack of mechanical loading and a lack of adequate mechanosensory ability or pro-inflammatory senescent markers occurs first during aging in osteocytes.

To our knowledge, there are no investigations directly assessing the influence of mechanical loading on osteocyte-related proteins in animal models of inflammatory conditions to determine the mechanical loading effects on osteocyte inflammatory changes. However, it is possible that in inflammatory pathologies, such as spinal cord injury where both chronic systemic inflammation and disuse are present, the inflammatory status with the lack of mechanical loading on osteocytes could exacerbate bone loss. Further work needs to be done on the interaction of inflammation and mechanical strains in osteocytes.

## Future Directions

With the accumulating knowledge of the role of osteocytes in inflammatory bone loss, future areas of interest may include therapeutically targeting osteocytes in inflammatory bone loss conditions. In inflammatory conditions, bone-specific treatments like bisphosphonates, anti-RANKL, and anti-sclerostin, all improve bone outcomes, but have no effect on inflammatory measures (96–98). Anti-inflammatory treatments, like anti-TNF, may improve bone mass (99), but potentially have negative side effects (100, 101). Therefore, viable treatments for inflammatory conditions are still needed. By directly impacting osteocytes via senolytic treatment or a JAK inhibitor in aged mice prevented the inflammatory senescent osteocyte phenotype (94). Additionally, in rodent models of inflammatory bowel disease, a soy protein diet and treatment with exogenous irisin decreased the inflammatory status of osteocytes and improved bone turnover (69, 102). Other anti-inflammatory treatments that improve bone in inflammatory conditions, like resolvin E1 (103), need to be examined for their impact on osteocytes. Furthermore, low-grade inflammation may be beneficial in some conditions like fracture healing (104). The role of other cytokines in bone physiology still needs to be elucidated as well as the relative contribution of osteocytic pro-inflammatory cytokines vs. those from other cell types in the marrow. Finally, the magnitude and duration of mechanical forces that could influence osteocyte-immune crosstalk has yet to be examined.

## Conclusions

Osteocytes play a central role in orchestrating changes in bone turnover. In this review, we present literature that supports an overlap between classical osteocyte regulatory proteins with known mechanosensory functions (RANKL, OPG, sclerostin, etc.) and immune factors that directly impact osteocytes and their communication with osteoblasts and osteoclasts. Pro-inflammatory signals stimulate osteocyte apoptosis, increase osteocyte cytokine production, and alter osteocytic proteins controlling bone turnover. Therefore, osteocytes are key players in inflammatory bone loss. This indicates that osteocytes may be targets for preventing/treating inflammatory bone alterations.

## Author Contributions

CM and SN drafted and edited the manuscript and approved the final version.

### Conflict of Interest Statement

The authors declare that the research was conducted in the absence of any commercial or financial relationships that could be construed as a potential conflict of interest.

## References

[1] Agrawal M Arora S Li J Rahmani R Sun L Steinlauf AF Bone, inflammation, and inflammatory bowel disease. Curr Osteoporos Rep. (2011) 2011:251–7. 10.1007/s11914-011-0077-9 21935582

[2] Attia EAS Khafagy A Abdel-Raheem S Fathi S Saad AA. Assessment of osteoporosis in psoriasis with and without arthritis: correlation with disease severity. Int J Dermatol. (2011) 50:30–5. 10.1111/j.1365-4632.2010.04600.x 21182499

[3] Haugeberg G Uhlig T Falch JA Halse JI Kvien TK. Bone mineral density and frequency of osteoporosis in female patients with rheumatoid arthritis. Arthritis Rheum. (2000) 43:522–30. 10.1002/1529-0131(200003)43:3<522::AID-ANR7>3.0.CO;2-Y 10728744

[4] Bultink IEM. Osteoporosis and fractures in systemic lupus erthyematosus. Arthritis Care Res. (2012) 64:2–8. 10.1002/acr.20568 22213721

[5] Bernstein CM Blanchard JF Leslie W Wadja A Yu BN. Incidence of fracture among patients with inflammatory bowel disease. Ann Intern Med. (2000) 133:795–9. 10.7326/0003-4819-133-10-200011210-00012 11085842

[6] Klaus J Armbrecht G Steinkamp M Brückel J Rieber A Adler G. High prevalence of osteoporotic vertebral fractures in patients with Crohn's disease. Gut. (2002) 51:654–8. 10.1136/gut.51.5.654 12377802 PMC1773437

[7] Van Staa TP Geusens P Bijlsma JWJ Leufkens HGM Cooper C. Clinical assessment of the long-term risk of fracture in patients with rheumatoid arthritis. Arthritis Rheum. (2006) 54:3104–12. 10.1002/art.22117 17009229

[8] Ogdie A Harter L Shin D Baker J Takeshita J Choi HK. The risk of fracture among patients with psoriatic arthritis and psoriasis: a population-based study. Ann Rheum Dis. (2017) 76:882–5. 10.1136/annrheumdis-2016-210441 28093419 PMC5384863

[9] Donath MY Shoelson SE. Type 2 diabetes as an inflammatory disease. Nature Rev Immunol. (2011) 11:98–107. 10.1038/nri2925 21233852

[10] Miyamoto T Carrero JJ Stenvinkel P. Inflammation as a risk factor and target for therapy in chronic kidney disease. Curr Opinion Nephrol Hypertens. (2011) 20:662–8. 10.1097/MNH.0b013e32834ad504 21825982

[11] Allison DJ Ditor DS. Immune dysfunction and chronic inflammation following spinal cord injury. Spinal Cord. (2015) 53:14–8. 10.1038/sc.2014.184 25366531

[12] Ginaldi L Di Benedetto MC De Martinis M. Osteoporosis, inflammation and ageing. Immun Ageing. (2005) 2:14. 10.1186/1742-4933-2-14 16271143 PMC1308846

[13] Okamoto K Nakashima T Shinohara M Negishi-Koga T Komatsu N Terashima A. Osteoimmunology: the conceptual framework unifying the immune and skeletal systems. Physiol Rev. (2017) 97:1295–349. 10.1152/physrev.00036.2016 28814613

[14] Turner MD Nedjai B Hurst T Pennington DJ. Cytokines and chemokines: at the crossroads of cell signalling and inflammatory disease. BBA-Mol Cell Res. (2014) 1843:2563–82. 10.1016/j.bbamcr.2014.05.014 24892271

[15] Redlich K Smolen JS. Inflammatory bone loss: pathogenesis and therapeutic intervention. Nature Rev. (2012) 11:234–50. 10.1038/nrd3669 22378270

[16] Kobayashi K Takahashi N Jimi E Udagawa N Takami M Kotake S. Tumor necrosis factor-α stimulates osteoclast differentiation by a mechanism independent of the ODF/RANKL-RANK interaction. J Exp Med. (2000) 191:275–85. 10.1084/jem.191.2.275 10637272 PMC2195746

[17] Lam J Takeshita S Barker JE Kanagawa O Ross FP Teitelbaum SL. TNF-α induces osteoclastogenesis by direct stimulation of macrophages exposed to permissive levels of RANK ligand. J Clin Invest. (2000) 106:1481–8. 10.1172/JCI11176 11120755 PMC387259

[18] Gowen M Wood DD Ihrie EJ McGuire MKB Russell GG. An interleukin 1 like factor stimulates bone resorption *in vitro*. Nature. (1983) 306:378–80. 10.1038/306378a0 6606132

[19] Pfeilschifter J Chenu C Bird A Mundy GR Roodman GD. Interleukin-1 and tumor necrosis factor stimulate the formation of human osteoclastlike cells *in vitro*. J Bone Miner Res. (1989) 4:113–8. 10.1002/jbmr.5650040116 2785743

[20] Nanes MS. Tumor necrosis factor-α: molecular and cellular mechanisms in skeletal pathology. Gene. (2003) 321:1–15. 10.1016/S0378-1119(03)00841-2 14636987

[21] Steeve KT Marc P Sandrine T Dominique H Yannick F IL-6, RANKL, TNF-alpha/IL-1: interrelations in bone resorption pathology. Cytokine Growth Factor Rev. (2004) 15:49–60. 10.1016/j.cytogfr.2003.10.005 14746813

[22] Hofbauer LC Lacey DL Dunstan CR Spelsberg TC Riggs BL Khosla S Interleukin-1β and tumor necrosis factor-α, but not interleukin-6, stimulate osteoprotegerin ligand gene expression in human osteoblastic cells. Bone. (1999) 25:255–9. 10.1016/S8756-3282(99)00162-3 10495128

[23] Hofbauer LC Dunstan CR Spelsberg TC Riggs BL Khosla S. Osteoprotegerin production by human osteoblast lineage cells is stimulated by vitamin D, bone morphogenetic protein-2, and cytokines. Biochem Biophys Res Comm. (1998) 250:776–81. 10.1006/bbrc.1998.9394 9784422

[24] Kotake S Udagawa N Takahashi N Matsuzaki K Itoh K Ishiyama S. IL-17 in synovial fluids from patients with rheumatoid arthritis is a potent stimulator of osteoclastogenesis. J Clin Invest. (1999) 103:1345–52. 10.1172/JCI5703 10225978 PMC408356

[25] Adamopoulos IE Chao C Geissler R Laface D Blumenschein W Iwakura Y. Interleukin-17A upregulates receptor activator of NF-κB on osteoclast precursors. Arthr Res Ther. (2010) 12:R29. 10.1186/ar2936 20167120 PMC2875663

[26] Abbas S Zhang YH Clohisy JC Abu-Amer Y. Tumor necrosis factor-α inhibits pre-osteoblast differentiation through its type-1 receptor. Cytokine. (2003) 22:33–41. 10.1016/S1043-4666(03)00106-6 12946103

[27] Bertolini DR Nedwin GE Bringman TS Smith DD Mundy GR Stimulation of bone resorption and inhibition of bone formation in vitro by human tumor necrosis factors. Nature. (1986) 319:516–8. 10.1038/319516a0 3511389

[28] Kaneki H Guo R Chen D Yao Z Schwarz EM Zhang YE. Tumor necrosis factor promotes Runx2 degradation through up-regulation of Smurf1 and Smurf2 in osteoblasts. J Biol Chem. (2006) 281:4326–33. 10.1074/jbc.M509430200 16373342 PMC2647592

[29] Scharla SH Strong DD Mohan S Chevalley T Linkhart TA. Effect of tumor necrosis factor-α on the expression of insulin-like growth factor I and insulin-like growth factor binding protein 4 in mouse osteoblasts. Eur J Endocrinol. (1994) 131:293–301. 10.1530/eje.0.1310293 7522842

[30] Pavalko FM Gerard RL Ponik SM Gallagher PJ Jin Y Norvell SM Fluid shear stress inhibits TNF-a-induced apoptosis in osteoblasts: A role for fluid shear stress-induced activation of PI3-kinase and inhibition of caspase-3. J Cell Physiol. (2002) 194:194–205. 10.1002/jcp.10221 12494458

[31] Stashenko P Dewhirst FE Rooney ML Desjardins LA Heeley JD Interleukin-1β is a potent inhibitor of bone formation *in vitro*. J Bone Miner Res. (1987) 2:559–65. 10.1002/jbmr.5650020612 3502684

[32] Bernardo ME Fibbe WE. Mesenchymal stromal cells: sensors and switchers of inflammation. Cell Stem Cell. (2013) 13:392–402. 10.1016/j.stem.2013.09.006 24094322

[33] Crotti TN Dharmapatni AA Alias E Haynes DR. Osteoimmunology: major and costimulatory pathway expression associated with chronic inflammatory induced bone loss. J Immunol Res. (2015) 2015:281287. 10.1155/2015/281287 26064999 PMC4433696

[34] Cheng J Liu J Shi Z Xu D Luo S Siegal GP. Interleukin-4 inhibits RANKL-induced NFATc1 expression via STAT6: A novel mechanism mediating its blockade of osteoclastogenesis. J Cell Biochem. (2011) 112:3385–92. 10.1002/jcb.23269 21751242 PMC3580163

[35] Evans KE Fox SW. Interleukin-10 inhibits osteoclastogenesis by reducting NFATc1 expression and preventing its translocation to the nucleus. BMC Cell Biol. (2007) 8:4. 10.1186/1471-2121-8-4 17239241 PMC1781937

[36] Fujii T Kitaura H Kimura K Hakami ZW Takano-Yamamoto T. IL-4 inhibits TNF-α-mediated osteoclast formation by inhibition of RANKL expression in TNF-α-activated stromal cells and direct inhibition of TNF-α-activated osteoclast precursors via a T-cell-independent mechanism *in vivo*. Bone. (2012) 51:771–80. 10.1016/j.bone.2012.06.024 22776139

[37] Xu LX Kukita T Kukita A Otsuka T Niho Y Iijima T. Interleukin-10 selectively inhibits osteoclastogenesis by inhibiting differentiation of osteoclast progenitors into preosteoclast-like cells in rat bone marrow culture system. Cell Physiol. (1995) 165:624–9. 10.1002/jcp.1041650321 7593242

[38] Stein NC Kreutzmann C Zimmermann SP Niebergall U Hellmeyer L Goettsch C. Interleukin-4 and interleukin-13 stimulate the csteoclast inhibitor osteoprotegerin by human endothelial cells through the STAT6 pathway. J Bone Miner Res. (2008) 23:750–8. 10.1359/jbmr.080203 18251702

[39] Dresner-Pollak R Gelb N Rachmilewitz D Karmeli F Weinreb M. Interleukin 10-deficient mice develop osteopenia, decreased bone formation, and mechanical fragility of long bones. Gastroenterol. (2004) 127:792–801. 10.1053/j.gastro.2004.06.013 15362035

[40] Bonewald LF. Osteocytes as dynamic multifunctional cells. Ann NY Acad Sci. (2007) 1116:281–90. 10.1196/annals.1402.018 17646259

[41] Bonewald LF. The amazing osteocyte. J Bone Miner Res. (2011) 26:229–38. 10.1002/jbmr.320 21254230 PMC3179345

[42] Li X Zhang Y Kang H Liu W Liu P Zhang J. Sclerostin binds to LRP5/6 and antagonizes canonical Wnt signaling. J Biol Chem. (2005) 280:19883–7. 10.1074/jbc.M413274200 15778503

[43] Ten Dijke P Krause C de tGorter DJJ Lowick CWGM van Bezooijen RL. Osteocyte-derived sclerostin inhibits bone formation: its role in bone morphogenetic protein and Wnt signaling. J Bone Joint Surg. (2008) 90:31–5. 10.2106/JBJS.G.01183 18292354

[44] Winkler DG Sutherland MK Geoghegan JC Yu C Hayes T Skonier JE. Osteocyte control of bone formation via sclerostin, a novel BMP antagonist. EMBO J. (2003) 22:6267–76. 10.1093/emboj/cdg599 14633986 PMC291840

[45] Nakashima T Hayashi M Fukunaga T Kurata K Oh-hora M Feng JQ. Evidence for osteocyte regulation of bone homeostasis through RANKL expression. Nat Med. (2011) 17:1231–4. 10.1038/nm.2452 21909105

[46] Xiong J Onal M Jilka RL Weinstein RS Manolagas SC O'Brien CA. Matrix-embedded cells control osteoclast formation. Nature Med. (2011) 17:1235–42. 10.1038/nm.2448 21909103 PMC3192296

[47] Xiong J Piemontese M Onal M Campbell J Goellner JJ Dusevich V. Osteocytes, not osteoblasts or lining cells, are the main source of the RANKL required for osteoclast formation in remodeling bone. PLoS ONE. (2015) 10:e0138189. 10.1371/journal.pone.0138189 26393791 PMC4578942

[48] Manolagas SC Parfitt AM For whom the bell tolls: Distress signals from long-lived osteocytes and the pathogenesis of metabolic bone disease. Bone. (2013) 54:272–82. 10.1016/j.bone.2012.09.017 23010104 PMC3574964

[49] Plotkin LI. Apoptotic osteocytes and the control of targeted bone resorption. Curr Osteoporos Rep. (2014) 12:121–6. 10.1007/s11914-014-0194-3 24470254 PMC3952244

[50] Bonewald LF Johnson ML. Osteocytes, mechanosensing and Wnt signaling. Bone. (2008) 42:606–15. 10.1016/j.bone.2007.12.224 18280232 PMC2349095

[51] Burger EH Klein-Nulend J Mechanotransduction in bone - role of the lacunocanalicular network. FASEB J. (1999) 13:S101–S112. 10.1096/fasebj.13.9001.s101 10352151

[52] Schaffler MB Cheung WY Majeska R Kennedy O. Osteocytes: Master orchestrators of bone. Calcif Tissue Int. (2014) 94:5–24. 10.1007/s00223-013-9790-y 24042263 PMC3947191

[53] Aguirre JI Plotkin LI Stewart SA Weinstein RS Parfitt AM Manolagas SC. Osteocyte apoptosis is induced by weightlessness in mice and precedes osteoclast recruitment and bone loss. J Bone Miner Res. (2006) 21:605–15. 10.1359/jbmr.060107 16598381

[54] Cabahug-Zuckerman P Frikha-Benayed D Majeska RJ Tuthill A Yakar S Judex S. Osteocyte apoptosis caused by hindlimb unloading is required to trigger osteocyte RANKL production and subsequent resorption of cortical and trabecular bone in mice femurs. J Bone Miner Res. (2016) 31:1356–65. 10.1002/jbmr.2807 26852281 PMC5488280

[55] Nefla M Holzinger D Berenbaum F Jacques C. The danger from within: alarmins in arthritis. Nature Rev Rheumatol. (2016) 12:669. 10.1038/nrrheum.2016.162 27733758

[56] Bidwell JP Yang J Robling AG. Is HMGB1 an osteocyte alarmin? J Cell Biochem. (2008) 103:1671–80. 10.1002/jcb.21572 17948903

[57] You L Temiyasathit S Lee P Kim CH Tummala P Yao W. Osteocytes as mechanosensors in the inhibition of bone resorption due to mechanical loading. Bone. (2008) 42:172–9. 10.1016/j.bone.2007.09.047 17997378 PMC2583402

[58] Plotkin LI Gortazar AR Davis HM Condon KW Gabilondo H Maycas M Inhibition of osteocyte apoptosis prevents the increase in osteocytic receptor activator of nuclear factor κB ligand (RANKL) but does not stop bone resorption or the loss of bone induced by unloading. J Biol Chem. (2015) 290:18934–42. 10.1074/jbc.M115.642090 26085098 PMC4521013

[59] Robling AG Niziolek PJ Baldridge LA Condon KW Allen MR Alam I. Mechanical stimulation of bone *in vivo* reduces osteocyte expression of Sost/sclerostin. J Biol Chem. (2008) 283:5866–75. 10.1074/jbc.M705092200 18089564

[60] Metzger CE Brezicha JE Elizondo JP Narayanan SA Hogan HA Bloomfield SA. Differential responses of mechanosensitive osteocyte proteins in fore- and hindlimbs of hindlimb-unloaded rats. Bone. (2017) 105:26–34. 10.1016/j.bone.2017.08.002 28782619

[61] Bakker AD Kulkarni RN Klein-Nulend J Lems WF IL-6 alters osteocyte signaling toward osteoblasts but not osteoclasts. J Dent Res. (2014) 93:394–9. 10.1177/0022034514522485 24492932

[62] Lau KHW Baylink DJ Zhou XD Rodriguez D Bonewald LF Li Z. Osteocyte-derived insulin-like growth factor I is essential for determining bone mechanosensitivity. Am J Physiol Endocrinol Metab. (2013) 305:E271–81. 10.1152/ajpendo.00092.2013 23715728

[63] Reijnders CMA Bravenboer N Tromp AM Blankenstein MA Lips P. Effect of mechanical loading on insulin-like growth factor-I gene expression in rat tibia. J Endocrinol. (2007) 192:131–40. 10.1677/joe.1.06880 17210750

[64] Ahuja SS Zhao S Bellido T Plotkin LI Jimenez F Bonewald LF. CD40 ligand blocks apoptosis induced by tumor necrosis factor-α, glucocorticoids, and etoposide in osteoblasts and the osteocyte-like cell line murine long bone osteocyte-Y4. Endocrinol. (2003) 144:1761–9. 10.1210/en.2002-221136 12697681

[65] Bakker AD da Silva VC Krishan R Bacabac RG Blaauboer ME Lin YC. Tumor necrosis factor-α and interleukin-1β modulate calcium and nitric oxide signaling in mechanically stimulated osteocytes. Arthritis Rheum. (2009) 60:3336–45. 10.1002/art.24920 19877030

[66] Tan SD Kuijpers-Jagtman AM Semeins CM Bronckers ALJJ Maltha JC Von den Hoff JW Fluid shear stress inhibits TNF-α induced osteocyte apoptosis. J Dent Res. (2006) 85:2006 10.1177/154405910608501006 16998129

[67] Morita M Iwasaki R Sato Y Kobayashi T Watanabe R Oike T. Elevation of pro-inflammatory cytokine levels following anti-resorptive drug treatment is required for osteonecrosis development in infectious osteomyelitis. Sci Rep. (2017) 7:46322. 10.1038/srep46322 28387378 PMC5384218

[68] Metzger CE Narayanan A Zawieja DC Bloomfield SA. Inflammatory bowel disease in a rodent model alters osteocyte protein levels controlling bone turnover. J Bone Miner Res. (2017b) 32:802–13. 10.1002/jbmr.3027 27796050

[69] Narayanan SA Metzger CE Bloomfield SA Zawieja DC. Inflammation-induced lymphatic architecture and bone turnover changes are ameliorated by irisin treatment in chronic inflammatory bowel disease. FASEB J. (2018) 32:4848–61. 10.1096/fj.201800178R 29596023 PMC6103167

[70] Kanaji A Caicedo MS Virdi AS Sumner DR Hallab NJ Sena K. Co-Cr-Mo alloy particles induce tumor necrosis factor alpha production in MLO-Y4 osteocytes: A role for osteocytes in particle-induced inflammation. Bone. (2009) 45:528–33. 10.1016/j.bone.2009.05.020 19497395 PMC2725206

[71] Liao C Cheng T Wang S Zhang C Jin L Yang Y. Shear stress inhibits IL-17A-mediated induction of osteoclastogenesis via osteocyte pathways. Bone. (2017) 101:10–20. 10.1016/j.bone.2017.04.003 28414140

[72] Pathak JL Bakker AD Luten FP Verschueren P Lems WF Klein-Nulend J. Systemic inflammation affects human osteocyte-specific protein and cytokine expression. Calcif Tissue Int. (2016) 98:596–608. 10.1007/s00223-016-0116-8 26887974

[73] Chhana A Pool B Callon KE Tay ML Musson D Naot D. Monosodium urate crystals reduce osteocyte viability and indirectly promote a shift in osteocyte function towards a proinflammatory and proresorptive state. Arthritis Res Ther. (2018) 20:208. 10.1186/s13075-018-1704-y 30201038 PMC6131786

[74] Viglietti AIP Benitez PCA Gentilini MB Velásquez LN Fossati CA Giambartolomei GH Brucella abortus invasion of osteocytes modulates connexin 43 and integrin expression and induces osteoclastogenesis via receptor activator of NF-κB ligand and tumor necrosis factor-alpha secretion. Infect Immun. (2015) 84:11–20. 10.1128/IAI.01049-15 26459511 PMC4694014

[75] Ormsby RT Solomon LB Yang D Crotti TN Haynes DR Findlay DM. Osteocytes respond to particles of clinically-relevant conventional and cross-linked polyethylene and metal alloys by up-regulation of resorptive and inflammatory pathways. Acta Biomater. (2019) 87:296–306. 10.1016/j.actbio.2019.01.047 30690207

[76] Metzger CE Narayanan SA Zawieja DC Bloomfield SM A moderately elevated soy protein diet mitigates inflammatory changes in gut and in bone turnover during chronic TNBS-induced inflammatory bowel disease. Appl Physiol Nutr Met. (2018) 2018:23 10.1139/apnm-2018-0514 30352170

[77] Kulkarni RN Bakker AD Everts V Klein-Nulend J. Mechanical loading prevents the stimulating effect of IL-1β on osteocyte-modulated osteoclastogenesis. Biochem Biophys Res Comm. (2012) 420:11–6. 10.1016/j.bbrc.2012.02.099 22390927

[78] Li JY D'Amelio P Robinson J Walker LD Vaccaro C Luo T. IL-17A is increased in humans with primary hyperparathyroidism and mediates PTH-induced bone loss in mice. Cell Metabol. (2015) 22:799–810. 26456334 10.1016/j.cmet.2015.09.012 PMC4635034

[79] Wu Q Zhou X Huang D Yingchen JI Kang F. IL-6 enhances osteocyte-mediated osteoclastogenesis by promoting JAK2 and RANKL activity in vitro. Cell Physiol Biochem. (2017) 41:1360–9. 10.1159/000465455 28278513

[80] Graves DT Alshabab A Albiero ML Mattos M Corrêa JD Chen S. Osteocytes play an important role in experimental periodontitis in healthy and diabetic mice through expression of RANKL. J Clin Periodontol. (2018) 45:285–92. 10.1111/jcpe.12851 29220094 PMC5811370

[81] Kim JH Lee DE Cha JH Bak EJ Yoo YJ. Receptor activator of nuclear factor-κB ligand and sclerostin expression in osteocytes of alveolar bone in rats with ligature-induced periodontitis. J Peridontol. (2014) 85:e370–378. 10.1902/jop.2014.140230 25070541

[82] Kim JH Kim AR Choi YH Jang S Woo GH Cha JH. Tumor necrosis factor-α antagonist diminishes osteocytic RANKL and sclerostin expression in diabetes rats with periodontitis. PloS ONE. (2017) 12:e0189702. 10.1371/journal.pone.0189702 29240821 PMC5730195

[83] Heiland GR Zwerina K Baum W Kireva T Distler JH Grisanti M. Neutralisation of Dkk-1 protects from systemic bone loss during inflammation and reduces sclerostin expression. Ann Rheum Dis. (2010) 69:2152–9. 10.1136/ard.2010.132852 20858621

[84] Colditz J Thiele S Baschant U Garbe AI Niehrs C Hofbauer LC Osteogenic Dkk1 mediates glucocorticoid-induced but not arthritis-induced bone loss. J Bone Miner Res. (2019) 2019:3702 10.1002/jbmr.3702 30779862

[85] Shaw AT Maeda Y Gravallese EM. IL-17A deficiency promotes periosteal bone formation in a model of inflammatory arthritis. Arthr Res Ther. (2016) 18:104. 10.1186/s13075-016-0998-x 27165410 PMC4863346

[86] Lin C Jiang X Dai Z Guo X Weng T Wang J. Sclerostin mediates bone response to mechanical unloading through antagonizing Wnt/β-catenin signaling. J Bone Miner Res. (2009) 24:1651–61. 10.1359/jbmr.090411 19419300

[87] Baek K Hwang HR Park HJ Kwon A Qadir AS Ko SH. TNF-α upregulates sclerostin expression in obese mice fed a high-fat diet. Cell Physiol. (2014) 229:640–50. 10.1002/jcp.24487 24446199

[88] David V Martin A Isakova T Spaulding C Qi L Ramirez V. Inflammation and functional iron deficiency regulate fibroblast growth factor 23 production. Kidney Int. (2016) 89:135–46. 10.1038/ki.2015.290 26535997 PMC4854810

[89] Ito N Wijenayaka AR Prideaux M Kogawa M Ormsby RT Evdokiou A. Regulation of FGF23 expression in IDG-SW3 osteocytes and human bone by pro-inflammatory stimuli. Mol Cell Endocrinol. (2015) 399:208–18. 10.1016/j.mce.2014.10.007 25458698

[90] Uluçkan Ö Jimenez M Karbach S Jeschke A Gra-a O Keller J. Chronic skin inflammation leads to bone loss by IL-17-mediated inhibition of Wnt signaling in osteoblasts. Sci Transl Med. (2016) 8:330ra37. 10.1126/scitranslmed.aad8996 27089206

[91] Hemmatian H Bakker AD Klein-Nulend J van Lenthe GH. Aging, osteocytes, and mechanotransduction. Curr Osteopor Rep. (2017) 15:401–11. 10.1007/s11914-017-0402-z 28891009 PMC5599455

[92] Tiede-Lewis LM Dallas SL. Changes in the osteocyte lacunocanalicular network with aging. Bone. (2019) 122:103–13. 10.1016/j.bone.2019.01.025 30743014 PMC6638547

[93] Farr JN Fraser DG Wang H Jaehn K Ogrodnik MB Weivoda MM. Identification of senescent cells in the bone microenvironment. J Bone Miner Res. (2016) 31:1920–9. 10.1002/jbmr.2892 27341653 PMC5289710

[94] Farr JN Xu M Weivoda MM Monroe DG Fraser DG Onken JL Targeting cellular senescence prevents age-related bone loss in mice. Nature Med. (2017) 23:1072–82. 10.1038/nm.4385 28825716 PMC5657592

[95] Sherk VD Rosen CJ. Senescent and apoptotic osteocytes and aging: Exercise to the rescue? Bone. (2019) 121:255–8. 10.1016/j.bone.2019.02.006 30735796 PMC6459182

[96] Cohen SB Dore RK Lane NE Ory PA Peterfy CG Sharp JT. Denosumab treatment effects on structural damage, bone mineral density, and bone turnover in rheumatoid arthritis. Arthritis Rheum. (2008) 58:1299–309. 10.1002/art.23417 18438830

[97] Eddleston A Marenzana M Morre AR Stephens P Muzylak M Marshall D Robinson MK. A short treatment with an antibody to sclerostin can inhibit bone loss in an ongoing model of colitis. J Bone Miner Res. (2009) 24:1662–71. 10.1359/jbmr.090403 19419292

[98] Herrak P Görtz B Hayer S Redlich K Reiter E Gasser J. Zoledronic acid protects against local and systemic bone loss in tumor necrosis factor-mediated arthritis. Arthritis Rheum. (2004) 50:2327–37. 10.1002/art.20384 15248234

[99] Veerappan SG O'Morain CA Daly JS Ryan BM Review article: the effects of anti-tumour necrosis factor-α on bone metabolism in inflammatory bowel disease. Ailmentary Pharmcol Ther. (2011) 33:1261–72. 10.1111/j.1365-2036.2011.04667.x 21521250

[100] Atzeni F Talotta R Salaffi F Cassinotti A Varisco V Battellino M. Immunogenicity and autoimmunity during anti-TNF therapy. Autoimmunity Rev. (2013) 12:703–8. 10.1016/j.autrev.2012.10.021 23207283

[101] Ding T Deighton C Complications of Anti-TNF therapies. Future Med. (2007) 2:587–97. 10.2217/17460816.2.6.587

[102] Metzger CE Gong S Aceves M Bloomfield SA Hook MA. Osteocytes reflect a pro-inflammatory state following spinal cord injury in a rodent model. Bone. (2019) 120:465–75. 10.1016/j.bone.2018.12.007 30550849

[103] El Kholy K Van Dyke TE Freire M Chen T. Resolvin E1 promotes bone preservation under inflammatory conditions. Front Immunol. (2018) 9:1300. 10.3389/fimmu.2018.01300 29946319 PMC6005849

[104] Chan JK Glass GE Ersek A Freidin A Williams GA Gowers K. Low-dose TNF augments fracture healing in normal and osteoporotic bone by up-regulating the innate immune response. EMBO Mol Med. (2015) 7:547–61. 10.15252/emmm.201404487 25770819 PMC4492816

